# Case Report: Durable complete response of advanced-stage hepatocellular carcinoma to DEB−TACE combined with lenvatinib and camrelizumab

**DOI:** 10.3389/fimmu.2025.1549675

**Published:** 2025-06-20

**Authors:** Baiguo Xu, Yufeng Cui, Ning Wang, Zhongsong Gao, Qing Ye, Huiling Xiang

**Affiliations:** ^1^ Department of Gastroenterology and Hepatology, Tianjin University Central Hospital, Tianjin, China; ^2^ Department of Gastroenterology and Hepatology, Tianjin Third Central Hospital, Tianjin, China; ^3^ Department of Gastroenterology and Hepatology, Tianjin Institute of Hepatobiliary Disease, Tianjin, China; ^4^ Tianjin Key Laboratory of Extracorporeal Life Support for Critical Diseases, Tianjin University Central Hospital, Tianjin, China; ^5^ Department of Gastroenterology and Hepatology, Artificial Cell Engineering Technology Research Center, Tianjin, China; ^6^ Department of Radiology, Tianjin University Central Hospital, Tianjin, China

**Keywords:** hepatocellular carcinoma, locoregional therapy, drug eluting beads-TACE, immunotherapy, lenvatinib, camrelizumab

## Abstract

**Background:**

Hepatocellular carcinoma (HCC) with lung metastases is associated with a poor prognosis due to limited effective treatment options. Emerging evidence suggests that combining locoregional therapy, multi-kinase inhibitors (MKIs), and immune checkpoint inhibitors (ICIs) offers promising results for advanced HCC. However, the efficacy of innovative combinations of MKIs and ICIs remains inconclusive. Herein, we present a case of a patient with massive HCC and lung metastases, complicated with decompensated hepatitis B cirrhosis, who achieved complete remission (CR) lasting for 10 months following treatment with lenvatinib (an MKI), camrelizumab (a PD-1 inhibitor), and locoregional therapy.

**Case summary:**

A 58-year-old male patient with decompensated hepatitis B-induced liver cirrhosis and advanced HCC with lung metastases underwent drug-eluting bead transarterial chemoembolization (DEB-TACE) therapy. Initially, he received apatinib in combination with camrelizumab; however, due to intolerance to apatinib’s side effects, the regimen was adjusted to lenvatinib and camrelizumab. After three DEB-TACE sessions, 14 weeks of lenvatinib, and a 5-month course of camrelizumab, the patient achieved CR, with no tumor recurrence observed over 10 months of follow-up.

**Conclusion:**

The combination of DEB-TACE, lenvatinib, and camrelizumab demonstrated efficacy in a patient with advanced HCC and lung metastases. These findings suggest that integrating MKIs and ICIs may represent a potential treatment approach for select advanced HCC cases, warranting further validation in larger studies.

## Introduction

Primary liver cancer often remains asymptomatic in its early stages, leading to delayed diagnosis at advanced stages (1). Lung metastasis is the most frequent site of extrahepatic spread in hepatocellular carcinoma (HCC), with an incidence of 39.5%–55% (2–4). The 1-year overall survival (OS) rate for primary liver cancer patients with lung metastasis is approximately 10% (5, 6), with a median survival time of 4.5 months (7).

The standard treatment for HCC with lung metastasis (Barcelona Clinic Liver Cancer [BCLC] stage C) is systemic therapy (8, 9). Recent research has demonstrated that combining locoregional therapies with systemic treatment can improve objective response rates (ORR) and OS. However, the most effective systemic treatment combination for lung-metastatic HCC remains contentious, and treatment strategies are often tailored based on individual clinical experience. Drug-eluting beads TACE(DEB-TACE): this embolization strategy uses microspheres to block tumor blood supply while serving as carriers for chemotherapy drugs, enabling their slow and continuous release (10). Targeted therapies, through anti-angiogenesis mechanisms, may normalize tumor vasculature, enhance T-cell infiltration, downregulate regulatory T cells (Tregs), and improve the tumor microenvironment for immunotherapy (11, 12). Lenvatinib exerts antitumor effects by inhibiting multiple signaling pathways, including VEGFR, FGFR, and PDGFR simultaneously (13). Immune checkpoint inhibitors (ICIs) have shown promise in preventing HCC progression, recurrence, and metastasis (14).

Herein, we report a case of a patient with massive HCC, lung metastasis, and decompensated hepatitis B cirrhosis, who achieved sustained CR with progression-free survival for 13 months following treatment with DEB-TACE, lenvatinib, and camrelizumab.

## Case presentation

**Figure 1:**
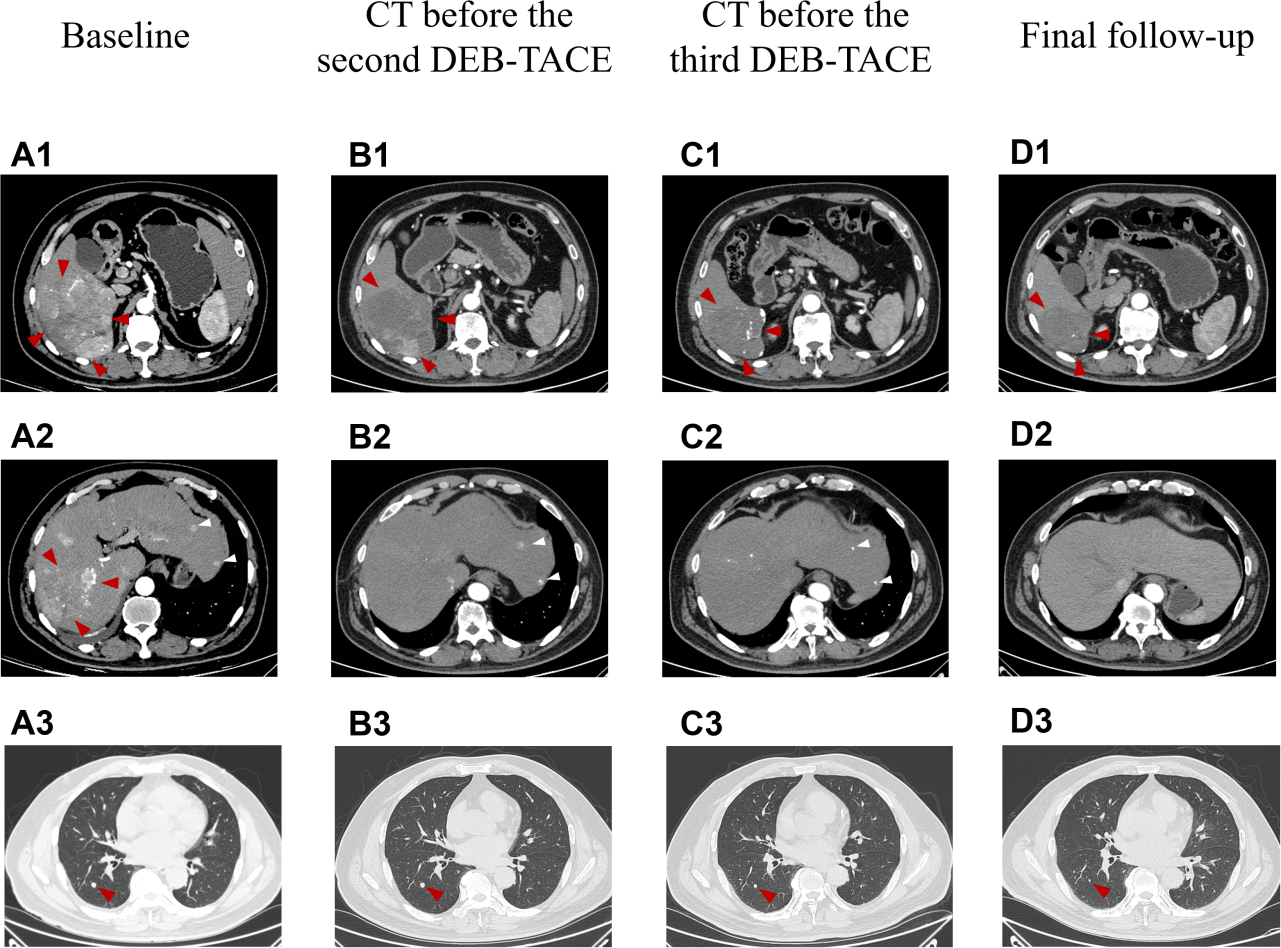
The representative chest and abdominal enhanced CT images changed during clinical treatment. **(A1–A3)** Before the first DEB-TACE treatment (at baseline), the abdomen enhanced CT showed a large lumpy blood-rich tumor in the right lobe of the liver, with a size of about 11.1 cm × 10.2 cm (shown by red arrows), multiple subfocies of HCC were also seen in the liver (shown by white arrows), and metastatic lesions in the dorsal segment of the lower lobe of the patient’s right lung (size about 0.7 cm × 0.7 cm); **(B1, B2)** Two months after the first DEB-TACE treatment (before the second TACE treatment), the massive tumor in the right lobe of the liver shrank (about 9.2 cm × 7.8 cm in size), and a large area of necrosis was visible in it, which was assessed as PR according to the mRECIST criteria (red arrows), and good iodized oil deposition was seen in the subfocies (white arrows); **(C1, C2)** Two months after the second DEB-TACE treatment (before the third TACE treatment), the original right lobe of the hepatic massive tumor (shown by red arrows) and intrahepatic subfocies (shown by white arrows) were inactive, and were assessed as CR according to mRECIST criteria; **(D1, D2)** Fourteen months after the first treatment (the final follow-up), the original hepatocellular carcinoma lesions in the liver were stable and inactive. **(B3–D3)** Two months after the first DEB-TACE treatment, two months after the second DEB-TACE treatment and 14 months after the first treatment.Chest CT images, the diameter of the dorsal nodule in the right lower lobe, 7mm, 6mm, 2mm, respectively. mRECIST, the Modified Response Evaluation Criteria in Solid Tumors.

A 58-year-old male patient with a history of hepatitis B, type 2 diabetes mellitus, and hypertension was admitted to our hospital in October 2023 with intermittent right upper abdominal pain lasting for 1 week. No family history of hereditary liver disease or cancer was documented. The patient denied smoking history and reported occasional light alcohol consumption (≤50 mL/month). No significant psychosocial stressors were identified. Chest and abdominal contrast-enhanced CT scans revealed a large mass (11.1 × 10.2 cm) with a rich blood supply in the right posterior hepatic lobe and multiple liver lesions, consistent with hepatocellular carcinoma (**Figures 1A1, A2**). Perihepatic effusion, small pulmonary nodules suggestive of metastases (**Figure 1A3**), and minimal right pleural effusion were also noted. The BCLC stage was classified as C.

Laboratory investigations showed normal complete blood counts and coagulation profiles. Liver function tests revealed serum albumin of 43.8 g/L, alanine aminotransferase (ALT) of 91 U/L, aspartate aminotransferase (AST) of 55 U/L, alkaline phosphatase of 131 U/L, gamma-glutamyl transpeptidase of 69 U/L, and total bilirubin of 14.8 µmol/L. The international normalized ratio (INR) was 0.98, while alpha-fetoprotein (AFP) was markedly elevated at 1.099.00 ng/mL. Hepatitis B viral DNA levels were 8.84 × 10^4^ IU/mL. Antiviral therapy with tenofovir disoproxil fumarate was initiated.

**Figure 2:**
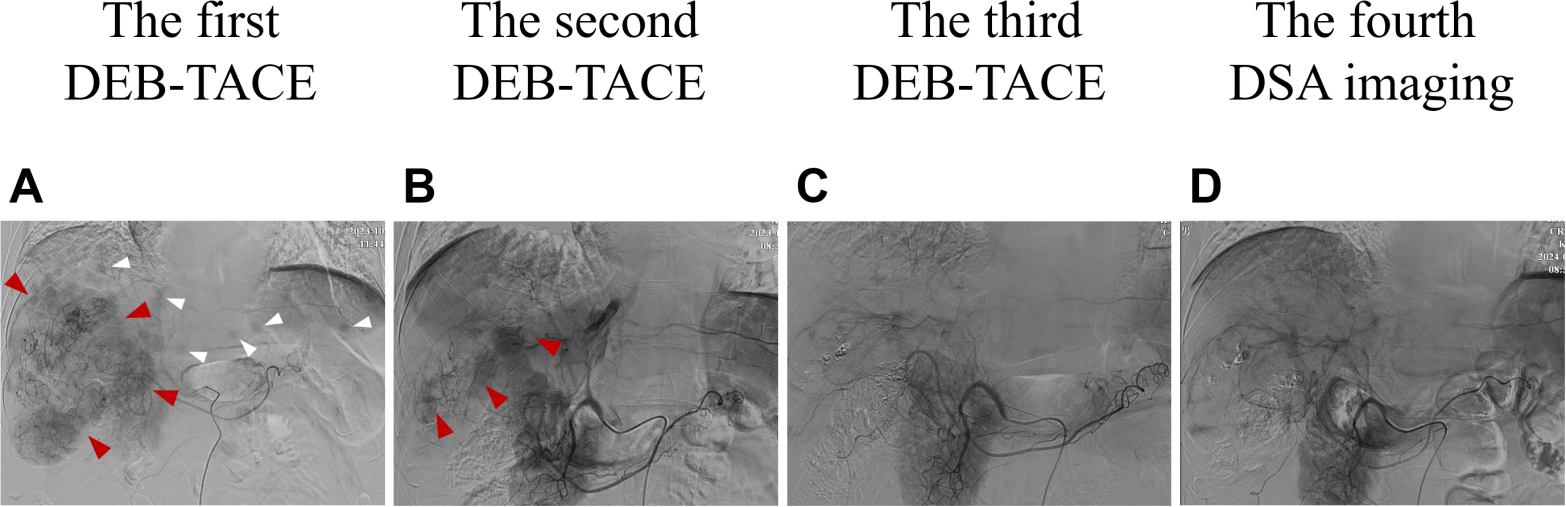
Changes in imaging of DSA during clinical treatment. **(A)** At baseline (the first DEB-TACE treatment), the DSA image was consistent with the enhanced CT scan: a large mass vascular-rich tumor with a size of about 11.1 cm × 10.2 cm was visible in the right lobe of the liver (shown by the red arrows), and multiple subfocies were also visible (shown by the white arrows). **(B)** Two months after the first DEB-TACE treatment (the second TACE treatment), the second DSA showed that the staining of the right lobe of the hepatic giant tumor was significantly reduced (about 9.2 cm ×7.8 cm in size), and there was no obvious staining in the intrahepatic subfocies. **(C)** Two months after the second DEB-TACE treatment (the third TACE treatment), the third DSA showed that there was no tumor staining in the original right lobe of the giant tumor and intrahepatic subfocies. **(D)** Two months after the third DEB-TACE treatment, the fourth DSA imaging also showed no tumor staining. DSA, digital artery angiography.

ULP (Ultra-Lipiodol, Guerbet, France) was used as the embolization agent, mixed with epirubicin for sustained drug delivery. The patient underwent DEB-TACE using 30 mg of epirubicin-loaded microspheres (1 g; diameter 300–500 μm; **Figure 2A**), without platinum compounds and was initially treated with apatinib and camrelizumab (200 mg every 3 weeks). However, Apatinib was discontinued due to grade 3 hand-foot skin reaction and hypertension. Lenvatinib (12 mg daily) was selected as an alternative due to its proven efficacy in HCC with preserved Child-Pugh A and lower incidence of dermatological toxicity. At 6 weeks post-treatment, a partial response (PR) was assessed based on the modified Response Evaluation Criteria in Solid Tumors (mRECIST; **Figures 1B1, B2**). The patient continued DEB-TACE using ULP (4.0 mL) and drug-loaded microspheres (40 mg epirubicin, 0.4 mL; **Figure 2B**). Follow-up abdominal contrast-enhanced CT scans at the 8th week until subsequent long-term follow-ups at 48 weeks confirmed changes consistent with TACE and immunotherapy (LR-TR Nonviable). Digital subtraction angiography (DSA) indicated no activity in the primary liver lesion, with no new tumor lesions identified. Complete response (CR) was confirmed using mRECIST criteria (**Figures 1C1, C2**, **2C**, **1D1, D2**, **2D**). Regular imaging follow-ups were conducted every 8 weeks. By December 2, 2024, chest and abdominal contrast-enhanced CT scans showed significant reduction in lung metastases and inactive hepatic malignant lesions (LR-TR Nonviable; **Figures 1D1–D3**).

**Figure 3:**
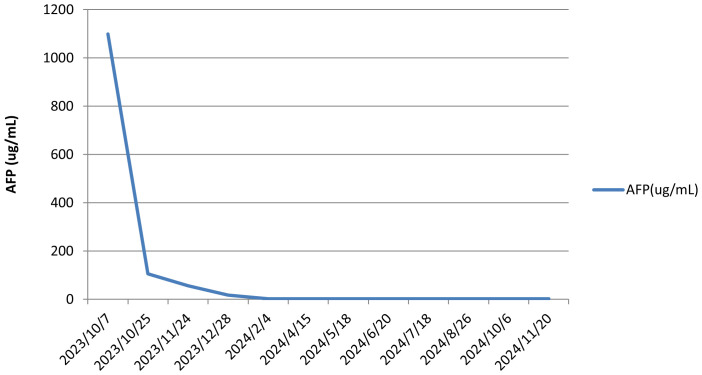
Changes in the AFP level during treatment. APF, alpha-fetoprotein.

The patient, a farmer with limited healthcare access, demonstrated consistent adherence to therapy, reflecting strong motivation for treatment. The patient continued treatment with lenvatinib and camrelizumab until December 2024, with chest and abdominal contrast-enhanced CT scans performed every 3–6 months. As of December 2, 2024, the patient had maintained complete remission for approximately 10 months with no tumor recurrence (**Figures 1D1, D2**). Lung metastatic lesions continued to shrink during this period (**Figures 1B3–D3**). AFP levels normalized, and HBV DNA decreased to <20 IU/mL. The patients had good compliance with the intervention measures and were regularly followed up. Mild fatigue (grade 1) and diarrhea (grade 1) were managed with supportive care (e.g., oral rehydration, loperamide). No treatment interruptions were required, demonstrating good tolerability. The AFP trend was shown in (**Figure 3**). The treatment timeline is summarized in **Table 1**.

**Table 1:** Treatment timeline of the patient.

Date	Locoregional therapy or ICIs	MKIs	(mRECIST)
2023.10.9	TACE		
2023.10.25	C	A	
2023.11.2		L	
2023.11.30	TACE	L	PR
2023.12.14	C		
2024.1.3	C		
2024.1.23	C		
2024.2.6	TACE		CR
2024.2.26	C		
2024.3.20	C		
2024.4.18	TACE		CR
2024.5.7	C		
2024.6.3	C		
2024.7.23	C		CR
2024.8.21	C		
2024.9.20	C		
2024.10.20	C		
2024.12.2	C		CR

MKIs, multi-kinase inhibitors; ICIs, immune checkpoint inhibitors; mRECIST, the modified Response Evaluation Criteria in Solid Tumors; TACE, transcatheter arterial chemoembolization; C, Camrelizumab; A, Apatinib; L, Lenvatinib; PR, partial response; CR, complete response.

The patient undergoes chest CT and contrast-enhanced CT every 8 weeks, and serum AFP testing monthly. Liver function and viral load (HBV DNA) are monitored quarterly. Given the risk of micrometastases, lenvatinib (12 mg/day) and camrelizumab (200 mg/q3w) will be continued for at least 12 months after achieving CR. In the event of disease progression, second-line therapies such as regorafenib or immune checkpoint inhibitor monotherapy will be considered, based on BCLC guidelines.

## Discussion

HCC often presents insidiously, with most patients being ineligible for surgical resection at diagnosis (15, 16). In mainland China, 55% of HCC diagnoses are at BCLC stage C, associated with poor prognosis (17). For these patients, systemic therapy remains the primary treatment modality (18). Approved systemic agents include TKIs and ICIs. Studies suggest a synergistic antitumor effect between TKIs and ICIs (19), although locoregional therapies such as TACE are not typically recommended for BCLC stage C HCC. TACE, including DEB-TACE, is a primary option for patients with BCLC stage B HCC (20). Shi et al. demonstrated superior efficacy and survival outcomes with DEB-TACE compared to cTACE in BCLC stage A and B patients (21). Meta-analyses also support better tumor response with DEB-TACE (22, 23). However, evidence supporting its benefit in BCLC stage C HCC remains limited, lacking multicenter randomized controlled trials.

TACE induces hepatic hypoxia, triggering vascular endothelial growth factor (VEGF) expression. TKIs inhibit VEGF, reversing immunosuppression caused by hypoxia and promoting antitumor immunity, enhancing the efficacy of ICIs. Tumor necrosis exposes tumor antigens, induces immunogenic cell death, and increases PD-L1 expression. ICIs block PD-1/PD-L1 interactions, alleviating immunosuppression and enhancing T-cell and antigen-presenting cell activity, ultimately inducing tumor cell death (24–26). Local therapy, TKIs, or ICIs alone are insufficient for robust antitumor effects. Therefore, combining locoregional therapy (DEB-TACE), TKIs, and ICIs appears rational for treating advanced HCC. To the best of our knowledge, this is the first reported case of a patient with extensive hepatocellular carcinoma, including intrahepatic multiple lesions and lung metastases, achieving long-term complete response through DEB-TACE combined with lenvatinib and camrelizumab.

This case underscores the critical importance of combining locoregional therapy, TKIs, and ICIs to manage HCC with intrahepatic massive tumors, multiple intrahepatic lesions, and lung metastases. Following DEB-TACE, the patient’s treatment was adjusted to a combination of lenvatinib and camrelizumab due to apatinib-induced side effects. This strategic adjustment not only demonstrates the adaptability required in clinical practice but also offers a valuable reference for future therapeutic approaches in similar cases.

Since the pivotal REFLECT study in 2018, lenvatinib has been established as a first-line therapy for HCC, demonstrating non-inferiority to sorafenib with improved clinical outcomes. Compared with sorafenib, lenvatinib extended median OS by 1.3 months (13.6 vs. 12.3 months), achieved a higher objective response rate (ORR) (24.1% vs. 9.2%), and prolonged progression-free survival (PFS) (8.9 vs. 3.7 months) (27). Furthermore, in a Chinese population, lenvatinib extended median OS to 15.0 months and PFS to 8.4 months, as reported in another clinical trial (28). Mechanistically, studies indicate that lenvatinib suppresses monocyte and macrophage activity within tumors, enhances T lymphocyte activation, and modulates immune responses (29). Additional evidence suggests that TKIs such as lenvatinib may improve tumor immunosuppressive pathways, thereby enhancing the efficacy of ICIs (30). These findings support the rationale for combining lenvatinib with PD-1 inhibitors, such as camrelizumab, to achieve superior tumor control and prolonged survival in advanced HCC.

Camrelizumab, a PD-1 inhibitor developed in China, targets PD-1 on CD4+ and CD8+ T lymphocytes, B cells, and natural killer cells, effectively countering PD-1-mediated immunosuppression and preventing tumor immune escape. A study evaluating the combination of camrelizumab with apatinib in advanced liver cancer reported an ORR of 50% and a disease control rate (DCR) of 85.7% (31), highlighting its significant efficacy in intermediate and advanced HCC.

In this case, the patient demonstrated a partial response (PR) to the initial DEB-TACE treatment, as assessed by mRECIST criteria, and achieved a complete response (CR) following the addition of lenvatinib and camrelizumab. These outcomes reinforce the efficacy of combining DEB-TACE with lenvatinib and camrelizumab in treating HCC.

The CHANCE001 trial further substantiates the benefit of combining TACE with targeted and immunotherapy. Patients with intermediate-to-advanced HCC receiving this combination achieved significantly longer median OS (19.2 vs. 15.7 months) and PFS (9.5 vs. 8.0 months) compared with TACE alone (32). This regimen demonstrated strong anti-tumor effects in advanced HCC, achieving an ORR of up to 77.4% (30).

Despite these promising results, optimal strategies for selecting TACE modalities and combining TKIs with ICIs remain unresolved. This case highlights the success of an unconventional approach, involving DEB-TACE, camrelizumab, and regorafenib, in managing HCC with extrahepatic metastases. Such findings provide new insights and potential avenues for treating advanced cases. As a single-case report, these results require validation in multicenter cohorts to assess reproducibility. What is more, pre-treatment biomarker analysis (e.g., PD-L1) was unavailable, limiting mechanistic interpretation. Prospective studies incorporating pre-treatment biomarker analysis are warranted to optimize patient selection for combined locoregional and systemic therapies, as biomarker-driven approaches, such as PD-L1 staining or T-cell infiltration assessment, have shown promise in predicting response to immune checkpoint inhibitors in HCC (33). Assessments of PD-L1 staining, VEGFR, and T-cell infiltration are needed for therapeutic effect evaluation in future cases, which is missing in the patient we reported.

In conclusion, this case of hepatitis B cirrhosis complicated by massive HCC with lung metastases was effectively managed through locoregional therapy combined with multitarget TKIs and ICIs. Given the lack of consensus on immunotherapy regimens for massive HCC with lung metastases and the limited evaluation of DEB-TACE combined with lenvatinib and camrelizumab, our findings suggest that this combination offers a viable treatment option. Future studies should investigate various combination regimens to optimize patient outcomes and establish a comprehensive treatment framework for advanced HCC.

## Data Availability

The original contributions presented in the study are included in the article/**Supplementary Material**. Further inquiries can be directed to the corresponding author.
