# Supplementary figures and images for: Case Report: Durable complete response of advanced-stage hepatocellular carcinoma to DEB−TACE combined with lenvatinib and camrelizumab
Source: Front Immunol. 2025 Jun 20;16:1549675. doi: 10.3389/fimmu.2025.1549675 (PMC12226579; doi:10.3389/fimmu.2025.1549675)

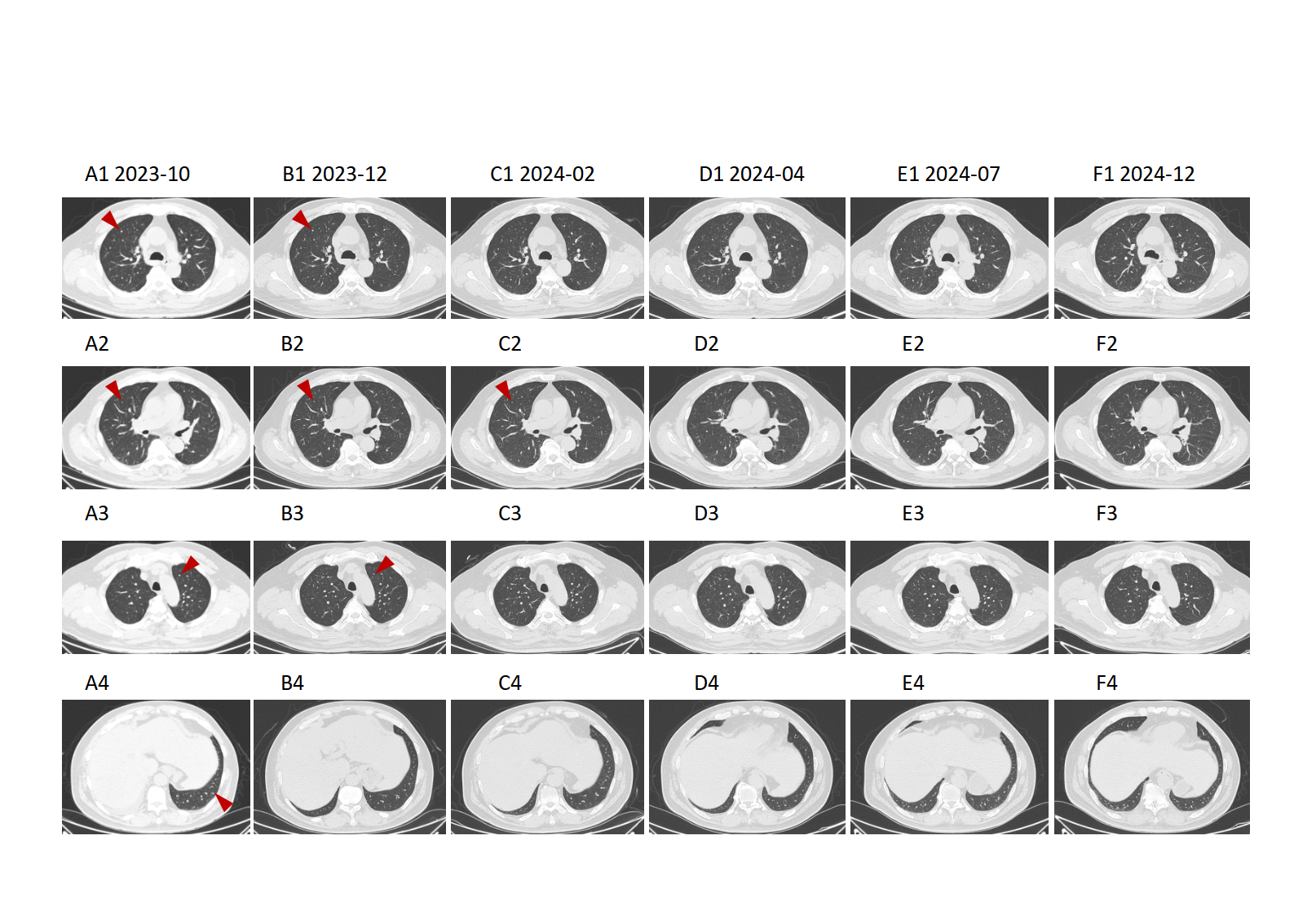

Supplement: Supplementary file 1 [file Image1.tif]
